# Review of guidelines on the treatment of metastatic renal cell carcinoma

**DOI:** 10.3747/co.v16i0.438

**Published:** 2009-05

**Authors:** D. Soulières

**Keywords:** Metastatic renal cell carcinoma, guidelines, therapies

## Abstract

Guidelines on the management of metastatic renal cell carcinoma (rcc) have emerged over the last couple of years because of the introduction of effective new targeted therapies. However, current guidelines are not always contemporary because of older data whose relevance is unclear in the context of the new therapies. Moreover, many of the relevant trials were interrupted prematurely because of significant advantages in progression-free survival. These circumstances sometimes make interpretation of the data and characterization of subgroups rather difficult. The current paper reviews the major guidelines available on the management of metastatic rcc and tries to put them into context.

## 1. INTRODUCTION

Principles that apply, or should apply, to the development of practice guidelines vary from one professional group to the next, based on their interpretation of the literature and on regional particularities that relate more to access to therapy than to applicability or true population differences. Therefore, a review of guidelines is *per se* an impossible endeavour, because it implies rendering judgment on an ensemble of processes that lead to a “best proposal” based on available data and current conditions.

Another impediment to a review of guidelines is the rapidity at which information regarding a particular pathology evolves. A magnitude of data with the potential to modify the practice of physicians caring for patients with metastatic renal cell carcinoma (rcc) has emerged over the last few years, including data from several studies stopped prematurely at interim analysis because of positive results. The interpretation of those data therefore depends on the premises that final results will concord with early analyses and that the statistical strength of the analyses of not only the primary endpoint, but also the secondary endpoints that should corroborate the primary endpoint, will stand. This approach is based on the Prentice principle, which states that a result has a higher probability of being truly positive if other related endpoints are also positive 1. An advantage in progression-free survival (pfs) has more chance of being true if a statistically significant advantage is also present for other aspects of efficacy such as response rate, time to treatment failure, and survival (if these data are available).

Remembering the Prentice principle, it will therefore be the prerogative of the editorial nature of the present paper to determine whether recommendations present in more than one guideline are likely to have a potential to affect practice. This paper also tries to comment on the reasons for the selection of certain treatment options and whether previous recommendations based on older data still apply in view of the treatment options that have so drastically modified the prognosis and survival of patients with metastatic rcc.

## 2. DISCUSSION

Recognizing the role of the von Hippel–Lindau gene product (vhl) and its value as a target in rcc has lent specific interest to this disease and to the controversy concerning which agent to use and when to use it 2. Moving from basically no efficacious therapy to several options also puts a strain on the deciders who have the responsibility to finance the new therapies to the best possible effect on an entire population of patients with rcc. Guidelines should therefore be as strong as possible and should reflect possibilities that have an effect not just on a selected trial population, but also on a general population. For example, the study conducted in British Columbia on all patients treated for rcc demonstrated that the introduction of sunitinib therapy, as compared with interferon therapy, for rcc resulted in a doubling of overall survival (os) based on historical results 3. These data reinforce the validity of guidelines proposing the use of sunitinib for metastatic rcc patients in the first line of therapy.

Internationally recognized guidelines that were evaluated for the purpose of the present paper include those produced by the European Association of Urology (eau), the U.S. National Comprehensive Cancer Network (nccn), and the Canadian Urological Association (cua—specifically, the Canadian Kidney Cancer Forum) 4–6. The eau publication dates from 2007, whereas the nccn guidelines were reviewed in January 2009. The published guidelines of the cua were published in 2008, but a revision has been completed, and its major elements are reported here.

### 2.1 On Surgery

All guidelines refer to data concerning the positive effect of nephrectomy on survival in patients with metastatic disease. Although all guidelines recommend surgery, some propose it only for patients for whom interferon therapy is scheduled or intended. Therefore, even though data pertaining to surgical therapy come from randomized trials, the systemic therapies in the comparator arms are not a standard that applies in 2009 7,8. Either restraining the applicability of surgery or questioning its validity or relevance for all patients is therefore adequate. The limited data on patients who did not undergo nephrectomy before a targeted systemic therapy was initiated do not seem to indicate a survival disadvantage. The data on the use of nephrectomy, a procedure with a potential for severe complications, should therefore be put into context and more fully studied, as is currently occurring in a trial by a French collaborative group.

### 2.2 On Prognostic Factors

Most trials of investigational agents in the last few years have been conducted using a stratification based on prognostic factors developed by Motzer and colleagues using a population of patients starting therapy with interferon 9. The effect of prognostic factors is extremely dependent on the time of measurement of those factors. Only in *post hoc* analyses can it be determined if the prognostic value of the factors still applies. Although validation efforts, based on data from recent trials, do not seem to support retention of much prognostic value for these prognostic factors, they have been used as a therapy selection criterion in all guidelines 10. Moreover, prognostic factors used for patient selection or stratification have varied from one study to another. This variation renders treatment selection based on biologic parameters and review of data more difficult, not to say a simple element of confusion.

Although data seem to indicate a variation in survival between poor-risk and other patients, the difference is not as significant as was initially reported by Motzer *et al.* based on their Memorial Sloan–Kettering experience with interferon patients. In view of this fact, the Canadian guidelines have recently been modified to partly account for this realization—at least for first-line therapy. As stated earlier, if guidelines are followed adequately, they should lead to a change in population-based statistics, such as those reported by Heng *et al.* 3.

Incoherence in patient definitions, such as that created by the addition of information on factors with uncertain relevance in the era of targeted therapy, makes evaluation of a whole-population effect difficult and perhaps impossible, because full and complete databases are not kept on all patients with rcc or other types of cancer. Definitions of cancer subgroups should therefore be based on tumour characteristics that should be sought only after prospective data demonstrate an effect on the efficacy of new therapies (not unlike the development of trastuzumab in breast cancer positive for the human epidermal growth factor receptor). The principle is therefore moving away from prognostic factors that should be independent of treatment and toward the identification of factors predictive of response to therapy.

Another option in designing and tailoring therapy is to use nomograms 11, but the use of nomograms in daily practice is not fully recognized, and validations based on population studies—such as the one performed by Heng and colleagues under Kollmannsberger—have not yet been reported.

### 2.3 On Anti-angiogenic Therapy

All guidelines state that sunitinib should be considered standard therapy in the first-line setting for metastatic rcc. The Canadian guidelines were recently modified to include all comers without regard for risk factors, because all subgroups analyzed demonstrated a benefit in terms of response and pfs. One element that strikes home in the analysis of guidelines and that probably led to these conclusions is the fact that sunitinib is the only vascular endothelial growth factor receptor (vegfr) tyrosine kinase inhibitor (tki) to be proposed in the first-line setting. In an attempt to demonstrate differences between that agent and sorafenib, the revision of the preferred surrogate markers of efficacy (that is, response rates) leads to the conclusion that the Prentice principle applies equally well to targeted therapy. Sunitinib demonstrated higher response rates in all studies, and the only possible assumption is that that finding correlates with an advantage in pfs, and ultimately os, as evidenced by multiple analyses of the phase iii trial and the paper by Heng *et al.* 3.

Tyrosine kinase inhibitors are not the only anti-angiogenic therapy that has proven active in metastatic rcc. A combination of bevacizumab and interferon is also accepted as an option in the guidelines, based on two trials that were reported prematurely because of an advantage in pfs. However, the primary endpoint of those trials was os, and a significant advantage there has not been reported at the time of writing. It therefore seems rather difficult to propose the bevacizumab and interferon combination as a standard in the first-line setting without formal evidence concerning the primary endpoint of the study. Moreover, important questions remain concerning the validity of this combination as compared with bevacizumab alone over interferon. The paucity of preclinical data leads most clinicians to believe that the combination will probably lead to more side effects than benefit. In fact, in the avoren [Avastin (Genentech, San Francisco, CA, U.S.A.) for Renal Cell Cancer] trial, the combination was less well tolerated than was interferon alone, whereas vegfr-tkis are generally better tolerated than interferon is. Because all guidelines list this combination treatment as an option, it should be so regarded, especially for patients in whom therapy with sunitinib is expected to possibly be a cause of significant grade 3 and 4 side effects.

Nonetheless, anti-angiogenic therapy is now the first and foremost choice in the initial management of metastatic rcc. Several new therapeutic options that are related to the mechanism of action of this agent are still under development, and the nccn guidelines still list—acceptably so—the inclusion of patients on clinical trial, especially if the trial permits comparison with the new standards of therapy. It is also worth mentioning that several groups have published proposed guidelines on the management of side effects specifically related to vegfr-tkis—more specifically, hypothyroidism, fatigue, palmoplantar erythema, and hypertension 12,13.

### 2.4 On Inhibitors of the Mammalian Target of Rapamycin

Mutations of the *VHL* gene occurring on both alleles provide an adequate explanation for the development of a targeted therapy based on a clonal anomaly that leads to an “oncogenic addiction.” However, other molecules involved in critical pathways of signal transduction seem to be particularly important in cell proliferation in rcc. The mammalian target of rapamycin molecule is closely related to, among other things, pathways associated with angiogenesis through the vhl complex 12.

Two molecules have been the object of trials and analyses in the context of guideline development: temsirolimus and everolimus. Unfortunately, establishing differences between these two molecules is difficult, because the trials studied very different populations in terms of patient characteristics and line of therapy. All guidelines propose the use of temsirolimus in the first line for poor-risk patients. But the “poor risk” defined in this population is different than that used as a stratification factor in trials evaluating anti-angiogenic agents. Moreover, as indicated earlier, the Canadian guidelines retained stratification for the vegfr-tkis, but accepted temsirolimus as an option for poor-risk patients.

Everolimus is the latest drug to be added to the list of therapeutic agents in rcc. First-line therapy has not been the subject of a trial, and in fact, most patients treated in further lines of therapy were in third, fourth, and fifth line with at least one vegfr-tki. Such a study was initiated when sunitinib was not accepted as a first-line standard. A true trial evaluating the efficacy of everolimus or another drug in the second-line setting after a vegfr-tki has not yet been performed. The revised Canadian guidelines recommend everolimus therapy as the standard after failure of a vegfr-tki; however, that recommendation does not specifically imply therapy immediately after failure of the vegfr-tki. It is noteworthy that the nccn does not list everolimus specifically, even though a clinical trial is listed as a consideration for patients with progression on a vegfr-tki.

### 2.5 On Periodic Review of Guidelines

The discovery that rcc is, in fact and contrary to previous data, a disease sensitive to targeted agents has made its therapy an area of intense and ongoing research activity. New results are expected to be presented at important oncology conferences in the coming years. Regarding existing guidelines, the review process is variable across groups. Intensification of research and availability of the resulting data should be expected to bring new and more effective therapies, and guidelines will and should help the medical community to differentiate the value of these therapies for the community of patients with metastatic rcc.

## 3. CONFLICT OF INTEREST

The author has no grant or affiliation to declare with respect to this paper.
